# Supplementary material for: Targeted intracellular delivery of molecular cargo to hypoxic human breast cancer stem cells
Source: bioRxiv. 2024 Nov 12:2024.01.12.575071. Preprint. [Version 2] doi: 10.1101/2024.01.12.575071 (PMC11601403; doi:10.1101/2024.01.12.575071)
Supplement: Supplement 2 [file media-2.pdf]

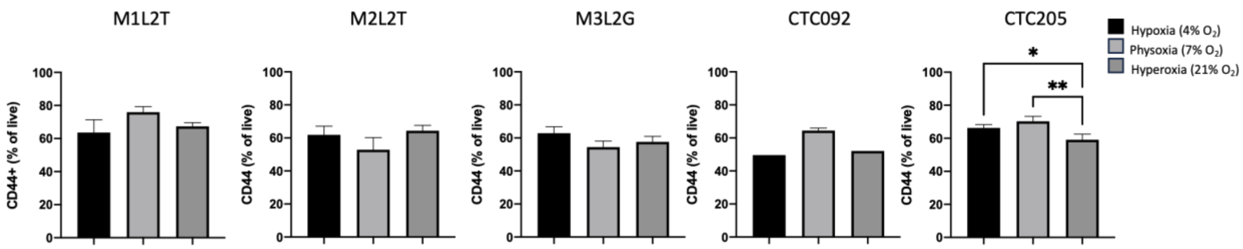

Supplemental Figure 1. CD44+ populations from each PDX line in different oxygen conditions. Percent CD44+ cells (from live) determined by flow cytometry for each PDX line used. Cells were maintained in hypoxia (4% O_2), physoxia (7% O_2) or hyperoxia (21% O_2). *p<.05, **p<.01.
