# Supplementary material for: Targeted intracellular delivery of molecular cargo to hypoxic human breast cancer stem cells
Source: bioRxiv. 2024 Nov 12:2024.01.12.575071. Preprint. [Version 2] doi: 10.1101/2024.01.12.575071 (PMC11601403; doi:10.1101/2024.01.12.575071)
Supplement: Supplement 4 [file media-4.pdf]

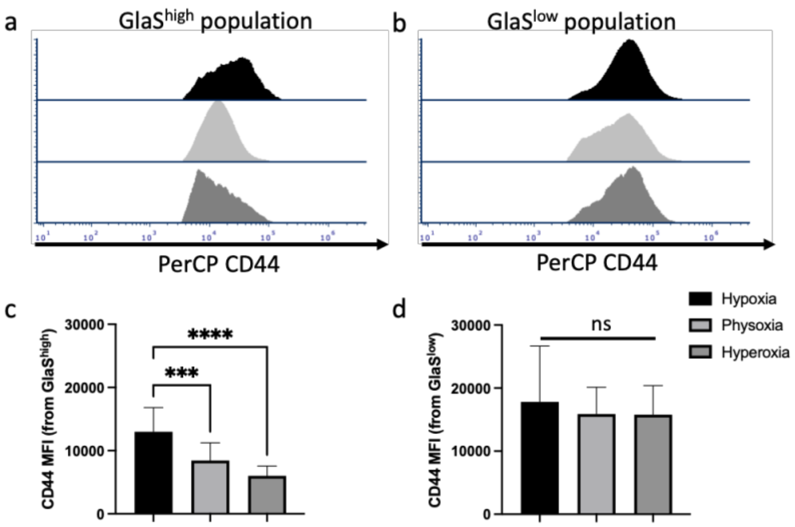

Supplemental Figure 1. CD44 expression is increased under hypoxia in GlaS^{high}small but not GlaS^{low}large population. Flow cytometry histograms demonstrate median fluorescence intensity (MFI) of PerCP CD44 for GlaS^{high}small (**a**) and GlaS^{low}large (**b**) populations. CD44 MFI was increased in cells cultured in hypoxic vs physoxic and hyperoxic conditions for GlaS^{high}small population (**c**). There was no significant difference in CD44 MFI under any oxygen condition for the GlaS^{low}large population. n=13 per O^2 concentration (n=5 PDX tumor types, n=1-3 technical replicates). ****p*<.001, *****p*<.0001, ns=not significant
