# Supplementary material for: Targeted intracellular delivery of molecular cargo to hypoxic human breast cancer stem cells
Source: bioRxiv. 2024 Nov 12:2024.01.12.575071. Preprint. [Version 2] doi: 10.1101/2024.01.12.575071 (PMC11601403; doi:10.1101/2024.01.12.575071)
Supplement: Supplement 5 [file media-5.pdf]

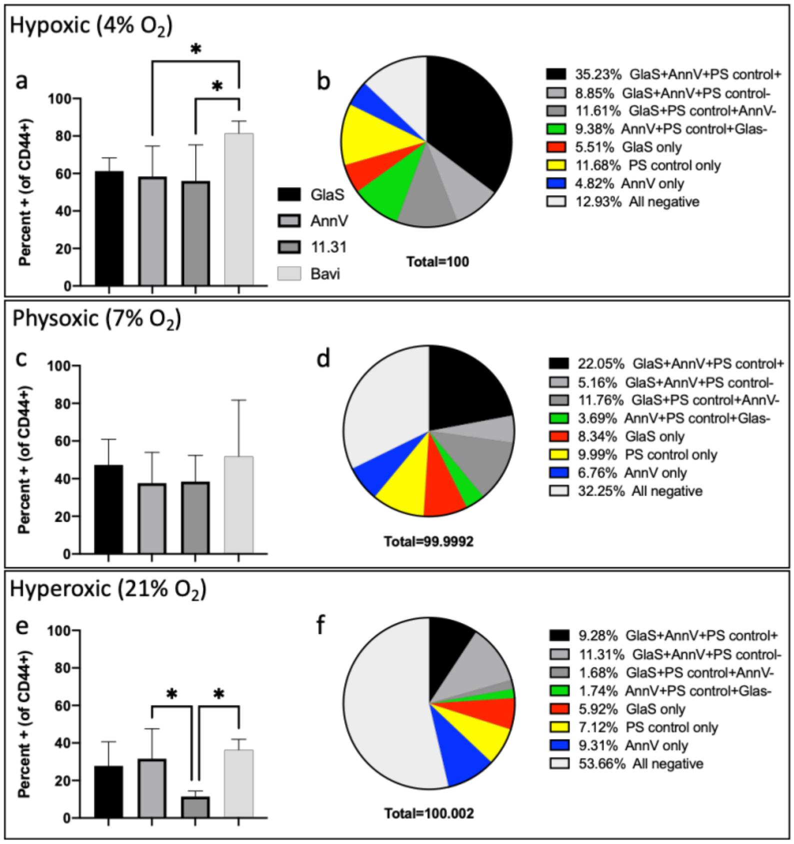


**Supplemental Figure 4 Differences in PS-binding GlaS, Annexin V and PS control Abs.** In hypoxic conditions, there was an increase in CD44+Bavi+ compared to CD44+AnnV+ and CD44+11.31+ cells (**a**). No difference in PS staining was observed for cells cultured under physoxic conditions (**c**). In hyperoxic conditions, CD44+11.31+ cells were decreased compared to CD44+AnnV+ and CD44+Bavi+ cells (**e**). Percent of cells with variations of GlaS, AnnV or PS control Ab staining are demonstrated for hypoxia (**b**), physoxia (**d**) and hyperoxia (**f**). AnnV; n=13 per $O^2$ concentration (n=5 PDX tumor types, n=1-3 technical replicates), Bavi/11.31; n=3-5 per $O^2$ concentration (n=3-5 PDX tumor types, n=1 technical replicate). **p*<.05
