# Supplementary material for: Targeted intracellular delivery of molecular cargo to hypoxic human breast cancer stem cells
Source: bioRxiv. 2024 Nov 12:2024.01.12.575071. Preprint. [Version 2] doi: 10.1101/2024.01.12.575071 (PMC11601403; doi:10.1101/2024.01.12.575071)
Supplement: Supplement 8 [file media-8.pdf]

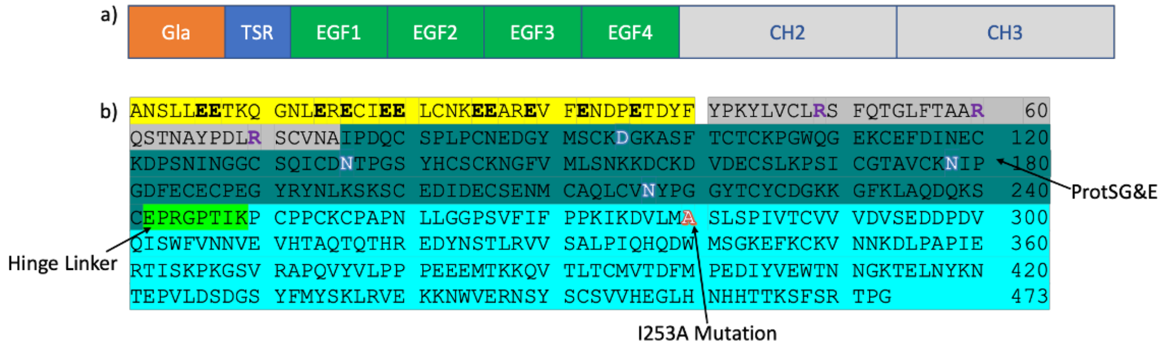

Supplemental Figure 7. GlaS protein domain structure and amino acid sequence. GlaS protein was derived from human Protein S and contains the Gla, TSR and four EGF-like domains from Protein S (ProtSG&E) and the murine IgG2A Fc domains CH2 and CH3 **(a).** Amino acid sequence of GlaS: Gla domain (yellow), TSR (grey), EGF domains (green), hinge linker (light green) and Fc domain containing the I253A substitution (light blue) **(b)**.
